# 9,10-Dioxoanthracene-1,4-diyl bis­(4-methyl­benzene­sulfonate)

**DOI:** 10.1107/S1600536812015814

**Published:** 2012-04-18

**Authors:** Thapong Teerawatananond, Chiaranan Kerdsamut, Sirirat Kokpol, Nongnuj Muangsin

**Affiliations:** a Research Centre for Bioorganic Chemistry, Department of Chemistry, Faculty of Science, Chulalongkorn University, Pathumwan, Bangkok 10330, Thailand; b Program of Bachelor of Science in Applied Chemistry (BSAC), Department of Chemistry, Faculty of Science, Chulalongkorn University, Pathumwan, Bangkok 10330, Thailand; c Computational Chemistry Unit Cell, Department of Chemistry, Faculty of Science, Chulalongkorn University, Pathumwan, Bangkok 10330, Thailand; d Center of Petroleum, Petrochemicals and Advanced Materials, Department of Chemistry, Faculty of Science, Chulalongkorn University, Pathumwan, Bangkok 10330, Thailand

## Abstract

The title mol­ecule, C_28_H_20_O_8_S_2_, has a T-shaped conformation. The central 9,10-anthraquinone moiety is bow-shaped with the two outer aromatic rings being inclined to one another by 13.99 (11)°. The benzenesulfonate rings are inclined to one another by 47.35 (12)°, and by 34.51 (11) and 17.88 (11)° to the bridging aromatic ring of the 9,10-anthraquinone moiety. In the crystal, C—H⋯O interactions link the mol­ecules into ribbons in [100].

## Related literature

For background to the structures of anthraquinones and their biological activity, see: Zielske (1987 ▶); Yatsenko *et al.* (2000 ▶); Huang *et al.* (2004 ▶); Meng *et al.* (2005 ▶); García-Sosa *et al.* (2006 ▶); Cho *et al.* (2006 ▶); Carland *et al.* (2010 ▶). For related structures, see: Swaminathan & Nigam (1967 ▶); Cao *et al.* (2007 ▶).
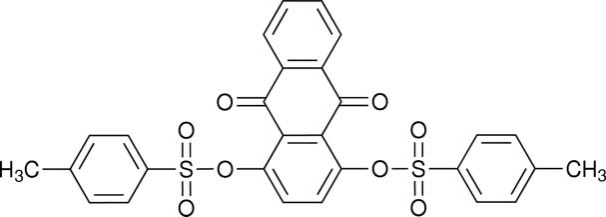


## Experimental

### 

#### Crystal data

C_28_H_20_O_8_S_2_
*M* *_r_* = 548.56
Triclinic, 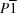

*a* = 9.6796 (2) Å
*b* = 10.9426 (3) Å
*c* = 13.1833 (4) Å
α = 111.122 (1)°
β = 90.961 (1)°
γ = 107.190 (1)°
*V* = 1232.41 (6) Å^3^
*Z* = 2
Mo *K*α radiation
μ = 0.27 mm^−1^
*T* = 296 K
0.35 × 0.20 × 0.20 mm

#### Data collection

Bruker APEXII CCD diffractometer
Absorption correction: multi-scan (*SADABS*; Bruker, 2008 ▶) *T*_min_ = 0.912, *T*_max_ = 0.948
12983 measured reflections
5616 independent reflections
3997 reflections with *I* > 2σ(*I*)
*R*_int_ = 0.031

#### Refinement

*R*[*F* ^2^ > 2σ(*F* ^2^)] = 0.044
*wR*(*F* ^2^) = 0.127
*S* = 1.02
5616 reflections
343 parameters
346 restraints
H-atom parameters constrained
Δρ_max_ = 0.28 e Å^−3^
Δρ_min_ = −0.30 e Å^−3^

### 

Data collection: *APEX2* (Bruker, 2008 ▶); cell refinement: *SAINT* (Bruker, 2008 ▶); data reduction: *SAINT*; program(s) used to solve structure: *SHELXS97* (Sheldrick, 2008 ▶); program(s) used to refine structure: *SHELXL97* (Sheldrick, 2008 ▶); molecular graphics: *ORTEP-3* (Farrugia, 1997 ▶); software used to prepare material for publication: *publCIF* (Westrip, 2010 ▶).

## Supplementary Material

Crystal structure: contains datablock(s) I, global. DOI: 10.1107/S1600536812015814/cv5268sup1.cif

Structure factors: contains datablock(s) I. DOI: 10.1107/S1600536812015814/cv5268Isup2.hkl

Supplementary material file. DOI: 10.1107/S1600536812015814/cv5268Isup3.cml

Additional supplementary materials:  crystallographic information; 3D view; checkCIF report

## Figures and Tables

**Table 1:** Hydrogen-bond geometry (Å, °)

*D*—H⋯*A*	*D*—H	H⋯*A*	*D*⋯*A*	*D*—H⋯*A*
C7—H7⋯O4^i^	0.93	2.48	3.333 (3)	153
C3—H3⋯O8^ii^	0.93	2.49	3.245 (3)	139

Symmetry codes: (i) 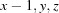; (ii) 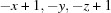.

**Fig. 1.**
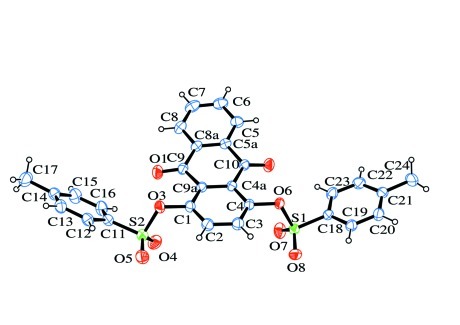
The molecular structure of the title compound showing the atom-numbering scheme. Displacement ellipsoids are drawn at the 50% probability level.

## supplementary crystallographic information

### Comment

Anthraquinone and its derivatives have been studies in the many fields, for example, antimicrobial and antibiotic activity (García-Sosa *et al.*, 2006), anticancer agents (Huang, *et al.*, 2004; Carland *et al.*, 2010). Additionally, both of unsubstituted and substituted anthraquinone play an important role in various photochemical and colorimetric sensor systems (Cho *et al.*, 2006). The natural extracts or synthetic anthraquinones have been used in the field of dyes and pigments (Meng *et al.*, 2005; Yatsenko *et al.*, 2000; Cao *et al.*, 2007).

1,4-Bis(hydroxy)anthraquinone is one of an important anthraquinone starting materials for preparation the various anthraquinone dyes and pigments (Zielske, 1987). In this work, we report the intermediate of an anthraquinone dye with the two symmetric tosylate substituents.

The molecular structure of 1,4-bis(tosyloxy)anthraquinone consisting of the two tosylate groups substituted at 1,4-positions of anthraquinone core, has a dragonfly-like conformation with the stranded 9,10-anthraquinone fragment. The anthraquinone plane is distorted by 0.1814 Å from the mean plane defined by 16 atoms becuase of the steric effect of two substitued tosyl groups. The O1 and O2 atoms were deviatated from the anthraquinone mean plane with the distances of -0.2736 (16) Å and -0.1467 (15) Å, respectively, which are respectively in the normal range for the distortion of oxyquinone reported for 1,4-bis(hydroxy)anthraquinone (Swaminathan *et al.*, 1967). Additionally, the moderate intermolecular hydrogen bonds of *sp^2^*C—H···O have been investigated between the hydrogen atom bound the aromatic carbon inside quinone ring, and the oxygen atom at the sulfonate group in the *p*-toluenesulfonate moiety as Figure 2. The distance of C(7)—H(7)···O(4) is 3.333 (3) Å and C(3)—H(3)···O(8) is 3.245 (3) Å that showed in the Table 1. In the crystal structure, non-classical intermolecular C—H···O hydrogen bonds link molecules into ribbons in [100].

### Experimental

1,4-Bis(tosyloxy)anthraquinone was prepared by a stirred solution of 1,4-bis(hydroxyl)anthraquinone or quinizarin (0.241 g, 1.00 mmol) in 25 mL of dry dichloromethane was added triethylamine (0.205 g, 2.03 mmol) and p-toluenesulfonyl chloride (0.383 g, 2.01 mmol). The solution was stirred at room temperature for 24 hours. The precipitate was filtered off and then washed with water and dried over magnesium sulfate. Filtration of slurry gave a bright yellow-green solid. The final product was recrystallized in hexane:dichloromethane using slow evaporation which was suitable for X-ray diffraction analysis. Additionally, ^1^H of 1,4-bis(tosyloxy)anthraquinone were recorded in CDCl_3_ solution on a Varian Mercury Plus 400 spectrometer. ^1^H NMR spectrum (δ, ppm): 7.94-8.02 (2H,m); 7.69-7.91 (6H,m); 7.45 (2H,s); 7.24-7.33 (4H,m); 2.35 (6H,s) (Zielske et al., 1987).

### Refinement

All H-atoms were geometrically positioned and refined using a riding model, with C—H = 0.93 Å (aromatic) and 0.96 Å (methyl), and *U*_iso_(H) = 1.2–1.5 *U*_eq_(C).

### Crystal data

**Table:**

C_28_H_20_O_8_S_2_	*Z* = 2
*M**_r_* = 548.56	*F*(000) = 568
Triclinic, *P*1	*D*_x_ = 1.478 Mg m^−^^3^
Hall symbol: -P 1	Mo *K*α radiation, λ = 0.71073 Å
*a* = 9.6796 (2) Å	Cell parameters from 3943 reflections
*b* = 10.9426 (3) Å	θ = 2.6–27.1°
*c* = 13.1833 (4) Å	µ = 0.27 mm^−^^1^
α = 111.122 (1)°	*T* = 296 K
β = 90.961 (1)°	Block, yellow–orange
γ = 107.190 (1)°	0.35 × 0.20 × 0.20 mm
*V* = 1232.41 (6) Å^3^	

### Data collection

**Table:**

Bruker APEXII CCD diffractometer	5616 independent reflections
Radiation source: fine-focus sealed tube	3997 reflections with *I* > 2σ(*I*)
Graphite monochromator	*R*_int_ = 0.031
φ and ω scans	θ_max_ = 27.5°, θ_min_ = 1.7°
Absorption correction: multi-scan (*SADABS*; Bruker, 2008)	*h* = −9→12
*T*_min_ = 0.912, *T*_max_ = 0.948	*k* = −14→14
12983 measured reflections	*l* = −17→16

### Refinement

**Table:**

Refinement on *F*^2^	Primary atom site location: structure-invariant direct methods
Least-squares matrix: full	Secondary atom site location: difference Fourier map
*R*[*F*^2^ > 2σ(*F*^2^)] = 0.044	Hydrogen site location: inferred from neighbouring sites
*wR*(*F*^2^) = 0.127	H-atom parameters constrained
*S* = 1.02	*w* = 1/[σ^2^(*F*_o_^2^) + (0.0564*P*)^2^ + 0.3809*P*] where *P* = (*F*_o_^2^ + 2*F*_c_^2^)/3
5616 reflections	(Δ/σ)_max_ < 0.001
343 parameters	Δρ_max_ = 0.28 e Å^−^^3^
346 restraints	Δρ_min_ = −0.30 e Å^−^^3^

### Special details

**Table:**

Geometry. All e.s.d.'s (except the e.s.d. in the dihedral angle between two l.s. planes) are estimated using the full covariance matrix. The cell e.s.d.'s are taken into account individually in the estimation of e.s.d.'s in distances, angles and torsion angles; correlations between e.s.d.'s in cell parameters are only used when they are defined by crystal symmetry. An approximate (isotropic) treatment of cell e.s.d.'s is used for estimating e.s.d.'s involving l.s. planes.
Refinement. Refinement of *F*^2^ against ALL reflections. The weighted *R*-factor *wR* and goodness of fit *S* are based on *F*^2^, conventional *R*-factors *R* are based on *F*, with *F* set to zero for negative *F*^2^. The threshold expression of *F*^2^ > σ(*F*^2^) is used only for calculating *R*-factors(gt) *etc*. and is not relevant to the choice of reflections for refinement. *R*-factors based on *F*^2^ are statistically about twice as large as those based on *F*, and *R*- factors based on ALL data will be even larger.

### Fractional atomic coordinates and isotropic or equivalent isotropic displacement parameters (Å^2^)

**Table:**

	*x*	*y*	*z*	*U*_iso_*/*U*_eq_	
C1	0.0724 (2)	−0.3607 (2)	0.30321 (17)	0.0455 (5)	
C2	0.2026 (2)	−0.2898 (2)	0.37289 (19)	0.0540 (5)	
H2	0.2612	−0.3377	0.3859	0.065*	
C3	0.2451 (2)	−0.1479 (2)	0.42300 (19)	0.0526 (5)	
H3	0.3337	−0.0992	0.4688	0.063*	
C4	0.1559 (2)	−0.07826 (19)	0.40504 (16)	0.0410 (4)	
C4A	0.0214 (2)	−0.14744 (19)	0.33781 (15)	0.0381 (4)	
C5	−0.3343 (2)	−0.0885 (3)	0.2748 (2)	0.0572 (6)	
H5	−0.3097	0.0060	0.3157	0.069*	
C5A	−0.2305 (2)	−0.1552 (2)	0.26945 (16)	0.0441 (4)	
C6	−0.4726 (3)	−0.1623 (3)	0.2197 (2)	0.0700 (7)	
H6	−0.5425	−0.1183	0.2251	0.084*	
C7	−0.5085 (3)	−0.3014 (3)	0.1565 (2)	0.0713 (7)	
H7	−0.6016	−0.3501	0.1173	0.086*	
C8	−0.4074 (3)	−0.3691 (3)	0.1510 (2)	0.0616 (6)	
H8	−0.4325	−0.4632	0.1084	0.074*	
C8A	−0.2675 (2)	−0.2962 (2)	0.20916 (17)	0.0457 (5)	
C9	−0.1623 (2)	−0.3716 (2)	0.20721 (17)	0.0465 (5)	
C9A	−0.0210 (2)	−0.29290 (19)	0.28346 (16)	0.0404 (4)	
C10	−0.0799 (2)	−0.0713 (2)	0.32586 (16)	0.0426 (4)	
C11	−0.0130 (2)	−0.7359 (2)	0.09847 (17)	0.0465 (5)	
C12	−0.0189 (3)	−0.8400 (2)	0.13582 (17)	0.0508 (5)	
H12	0.0487	−0.8251	0.1935	0.061*	
C13	−0.1266 (3)	−0.9662 (2)	0.08608 (19)	0.0571 (6)	
H13	−0.1306	−1.0367	0.1107	0.068*	
C14	−0.2286 (3)	−0.9906 (2)	0.00061 (19)	0.0575 (6)	
C15	−0.2168 (3)	−0.8849 (3)	−0.0367 (2)	0.0693 (7)	
H15	−0.2822	−0.9008	−0.0961	0.083*	
C16	−0.1114 (3)	−0.7576 (2)	0.0115 (2)	0.0619 (6)	
H16	−0.1063	−0.6875	−0.0139	0.074*	
C17	−0.3479 (3)	−1.1281 (3)	−0.0518 (2)	0.0844 (9)	
H17A	−0.3392	−1.1883	−0.0159	0.127*	
H17B	−0.3392	−1.1692	−0.1282	0.127*	
H17C	−0.4413	−1.1144	−0.0447	0.127*	
C18	0.3179 (2)	0.32113 (19)	0.50215 (16)	0.0406 (4)	
C19	0.4237 (2)	0.4068 (2)	0.59098 (17)	0.0491 (5)	
H19	0.4989	0.3777	0.6082	0.059*	
C20	0.4172 (3)	0.5357 (2)	0.65400 (19)	0.0552 (5)	
H20	0.4894	0.5940	0.7133	0.066*	
C21	0.3059 (2)	0.5800 (2)	0.6309 (2)	0.0533 (5)	
C22	0.2026 (3)	0.4927 (2)	0.5408 (2)	0.0627 (6)	
H22	0.1280	0.5223	0.5234	0.075*	
C23	0.2062 (2)	0.3635 (2)	0.4759 (2)	0.0568 (6)	
H23	0.1350	0.3061	0.4158	0.068*	
C24	0.2962 (3)	0.7192 (2)	0.7032 (3)	0.0808 (9)	
H24A	0.3768	0.7652	0.7615	0.121*	
H24B	0.2062	0.7065	0.7337	0.121*	
H24C	0.2995	0.7744	0.6603	0.121*	
O1	−0.19121 (19)	−0.49172 (16)	0.14755 (15)	0.0704 (5)	
O2	−0.04323 (17)	0.05362 (15)	0.36092 (15)	0.0641 (4)	
O3	0.03027 (16)	−0.50532 (13)	0.25827 (12)	0.0530 (4)	
O4	0.14116 (19)	−0.49740 (17)	0.09439 (14)	0.0711 (5)	
O5	0.24035 (19)	−0.58494 (17)	0.21518 (16)	0.0765 (5)	
O6	0.19989 (14)	0.06504 (13)	0.46365 (11)	0.0434 (3)	
O7	0.2846 (2)	0.12386 (17)	0.30826 (13)	0.0737 (5)	
O8	0.46239 (17)	0.14656 (16)	0.45212 (16)	0.0697 (5)	
S1	0.32770 (6)	0.15885 (5)	0.42096 (4)	0.04743 (15)	
S2	0.11835 (6)	−0.57384 (5)	0.16304 (5)	0.05089 (16)	

### Atomic displacement parameters (Å^2^)

**Table:**

	*U*^11^	*U*^22^	*U*^33^	*U*^12^	*U*^13^	*U*^23^
C1	0.0499 (11)	0.0367 (10)	0.0512 (12)	0.0144 (9)	0.0099 (9)	0.0180 (9)
C2	0.0514 (12)	0.0494 (12)	0.0672 (14)	0.0226 (10)	0.0022 (11)	0.0240 (11)
C3	0.0479 (12)	0.0505 (12)	0.0569 (13)	0.0156 (10)	−0.0032 (10)	0.0186 (10)
C4	0.0441 (10)	0.0368 (10)	0.0396 (10)	0.0109 (8)	0.0060 (8)	0.0135 (8)
C4A	0.0397 (10)	0.0376 (10)	0.0388 (10)	0.0116 (8)	0.0063 (8)	0.0173 (8)
C5	0.0518 (13)	0.0649 (14)	0.0626 (14)	0.0238 (11)	0.0065 (10)	0.0291 (12)
C5A	0.0416 (10)	0.0503 (11)	0.0449 (11)	0.0135 (9)	0.0056 (8)	0.0244 (9)
C6	0.0480 (13)	0.0900 (19)	0.0858 (19)	0.0270 (13)	0.0071 (12)	0.0454 (16)
C7	0.0408 (13)	0.0908 (19)	0.0818 (18)	0.0077 (13)	−0.0053 (12)	0.0438 (16)
C8	0.0501 (13)	0.0571 (14)	0.0672 (15)	0.0024 (11)	−0.0046 (11)	0.0246 (12)
C8A	0.0405 (10)	0.0500 (11)	0.0461 (11)	0.0073 (9)	0.0040 (8)	0.0236 (9)
C9	0.0465 (11)	0.0404 (11)	0.0471 (11)	0.0076 (9)	0.0052 (9)	0.0158 (9)
C9A	0.0403 (10)	0.0390 (10)	0.0413 (10)	0.0106 (8)	0.0073 (8)	0.0164 (8)
C10	0.0428 (11)	0.0412 (11)	0.0455 (11)	0.0130 (9)	0.0052 (8)	0.0188 (9)
C11	0.0506 (11)	0.0455 (11)	0.0435 (11)	0.0206 (9)	0.0048 (9)	0.0130 (9)
C12	0.0610 (13)	0.0517 (12)	0.0408 (11)	0.0234 (10)	0.0063 (10)	0.0148 (9)
C13	0.0756 (16)	0.0475 (12)	0.0499 (13)	0.0205 (11)	0.0191 (11)	0.0198 (10)
C14	0.0603 (14)	0.0511 (13)	0.0481 (13)	0.0153 (11)	0.0129 (10)	0.0061 (10)
C15	0.0736 (17)	0.0646 (15)	0.0578 (15)	0.0224 (13)	−0.0144 (12)	0.0108 (12)
C16	0.0776 (17)	0.0521 (13)	0.0565 (14)	0.0241 (12)	−0.0045 (12)	0.0195 (11)
C17	0.0779 (19)	0.0662 (17)	0.0752 (19)	0.0004 (14)	0.0121 (15)	0.0069 (14)
C18	0.0389 (10)	0.0387 (10)	0.0435 (10)	0.0094 (8)	0.0053 (8)	0.0174 (8)
C19	0.0446 (11)	0.0488 (12)	0.0517 (12)	0.0149 (9)	−0.0005 (9)	0.0173 (10)
C20	0.0555 (13)	0.0462 (12)	0.0507 (13)	0.0090 (10)	−0.0001 (10)	0.0097 (10)
C21	0.0504 (12)	0.0403 (11)	0.0685 (15)	0.0115 (9)	0.0218 (11)	0.0218 (10)
C22	0.0486 (13)	0.0565 (14)	0.0903 (18)	0.0228 (11)	0.0054 (12)	0.0318 (13)
C23	0.0488 (12)	0.0510 (13)	0.0645 (14)	0.0116 (10)	−0.0071 (10)	0.0194 (11)
C24	0.0789 (19)	0.0469 (14)	0.111 (2)	0.0224 (13)	0.0374 (17)	0.0217 (14)
O1	0.0628 (11)	0.0473 (9)	0.0759 (12)	0.0118 (8)	−0.0066 (9)	0.0008 (8)
O2	0.0574 (10)	0.0394 (8)	0.0900 (12)	0.0142 (7)	−0.0070 (8)	0.0204 (8)
O3	0.0605 (9)	0.0348 (7)	0.0639 (9)	0.0163 (7)	0.0168 (7)	0.0181 (7)
O4	0.0729 (11)	0.0681 (11)	0.0697 (11)	0.0077 (9)	0.0174 (9)	0.0350 (9)
O5	0.0566 (10)	0.0625 (11)	0.0955 (14)	0.0258 (8)	−0.0134 (9)	0.0091 (9)
O6	0.0448 (8)	0.0353 (7)	0.0423 (7)	0.0068 (6)	0.0057 (6)	0.0108 (6)
O7	0.1037 (14)	0.0592 (10)	0.0436 (9)	0.0113 (9)	0.0181 (9)	0.0147 (7)
O8	0.0432 (9)	0.0529 (9)	0.1090 (14)	0.0186 (7)	0.0147 (9)	0.0240 (9)
S1	0.0476 (3)	0.0408 (3)	0.0491 (3)	0.0111 (2)	0.0124 (2)	0.0141 (2)
S2	0.0477 (3)	0.0467 (3)	0.0563 (3)	0.0174 (2)	0.0067 (2)	0.0158 (2)

### Geometric parameters (Å, º)

**Table:**

C1—C2	1.379 (3)	C13—C14	1.381 (3)
C1—O3	1.399 (2)	C13—H13	0.9300
C1—C9A	1.401 (3)	C14—C15	1.387 (4)
C2—C3	1.375 (3)	C14—C17	1.507 (3)
C2—H2	0.9300	C15—C16	1.373 (3)
C3—C4	1.376 (3)	C15—H15	0.9300
C3—H3	0.9300	C16—H16	0.9300
C4—C4A	1.396 (3)	C17—H17A	0.9600
C4—O6	1.399 (2)	C17—H17B	0.9600
C4A—C9A	1.413 (3)	C17—H17C	0.9600
C4A—C10	1.500 (3)	C18—C19	1.379 (3)
C5—C6	1.370 (3)	C18—C23	1.380 (3)
C5—C5A	1.395 (3)	C18—S1	1.744 (2)
C5—H5	0.9300	C19—C20	1.376 (3)
C5A—C8A	1.385 (3)	C19—H19	0.9300
C5A—C10	1.484 (3)	C20—C21	1.375 (3)
C6—C7	1.376 (4)	C20—H20	0.9300
C6—H6	0.9300	C21—C22	1.378 (3)
C7—C8	1.380 (4)	C21—C24	1.510 (3)
C7—H7	0.9300	C22—C23	1.375 (3)
C8—C8A	1.394 (3)	C22—H22	0.9300
C8—H8	0.9300	C23—H23	0.9300
C8A—C9	1.484 (3)	C24—H24A	0.9600
C9—O1	1.206 (2)	C24—H24B	0.9600
C9—C9A	1.502 (3)	C24—H24C	0.9600
C10—O2	1.209 (2)	O3—S2	1.6126 (15)
C11—C12	1.383 (3)	O4—S2	1.4180 (17)
C11—C16	1.383 (3)	O5—S2	1.4140 (18)
C11—S2	1.740 (2)	O6—S1	1.6094 (14)
C12—C13	1.377 (3)	O7—S1	1.4153 (18)
C12—H12	0.9300	O8—S1	1.4185 (17)
			
C2—C1—O3	117.61 (18)	C13—C14—C17	121.2 (2)
C2—C1—C9A	122.05 (18)	C15—C14—C17	120.9 (2)
O3—C1—C9A	120.18 (18)	C16—C15—C14	122.0 (2)
C3—C2—C1	119.6 (2)	C16—C15—H15	119.0
C3—C2—H2	120.2	C14—C15—H15	119.0
C1—C2—H2	120.2	C15—C16—C11	118.5 (2)
C2—C3—C4	119.7 (2)	C15—C16—H16	120.7
C2—C3—H3	120.1	C11—C16—H16	120.7
C4—C3—H3	120.1	C14—C17—H17A	109.5
C3—C4—C4A	122.00 (18)	C14—C17—H17B	109.5
C3—C4—O6	117.29 (17)	H17A—C17—H17B	109.5
C4A—C4—O6	120.54 (17)	C14—C17—H17C	109.5
C4—C4A—C9A	118.59 (17)	H17A—C17—H17C	109.5
C4—C4A—C10	121.41 (17)	H17B—C17—H17C	109.5
C9A—C4A—C10	119.96 (17)	C19—C18—C23	120.68 (19)
C6—C5—C5A	119.9 (2)	C19—C18—S1	119.46 (16)
C6—C5—H5	120.0	C23—C18—S1	119.85 (16)
C5A—C5—H5	120.0	C20—C19—C18	119.4 (2)
C8A—C5A—C5	120.04 (19)	C20—C19—H19	120.3
C8A—C5A—C10	121.30 (18)	C18—C19—H19	120.3
C5—C5A—C10	118.63 (19)	C21—C20—C19	121.2 (2)
C5—C6—C7	120.3 (2)	C21—C20—H20	119.4
C5—C6—H6	119.8	C19—C20—H20	119.4
C7—C6—H6	119.8	C20—C21—C22	118.0 (2)
C6—C7—C8	120.4 (2)	C20—C21—C24	120.9 (2)
C6—C7—H7	119.8	C22—C21—C24	121.1 (2)
C8—C7—H7	119.8	C23—C22—C21	122.2 (2)
C7—C8—C8A	119.9 (2)	C23—C22—H22	118.9
C7—C8—H8	120.0	C21—C22—H22	118.9
C8A—C8—H8	120.0	C22—C23—C18	118.4 (2)
C5A—C8A—C8	119.3 (2)	C22—C23—H23	120.8
C5A—C8A—C9	121.30 (18)	C18—C23—H23	120.8
C8—C8A—C9	119.3 (2)	C21—C24—H24A	109.5
O1—C9—C8A	120.74 (19)	C21—C24—H24B	109.5
O1—C9—C9A	122.00 (19)	H24A—C24—H24B	109.5
C8A—C9—C9A	117.25 (17)	C21—C24—H24C	109.5
C1—C9A—C4A	117.97 (17)	H24A—C24—H24C	109.5
C1—C9A—C9	121.38 (17)	H24B—C24—H24C	109.5
C4A—C9A—C9	120.65 (17)	C1—O3—S2	116.74 (12)
O2—C10—C5A	119.98 (18)	C4—O6—S1	116.91 (11)
O2—C10—C4A	122.49 (18)	O7—S1—O8	118.31 (12)
C5A—C10—C4A	117.53 (17)	O7—S1—O6	107.69 (9)
C12—C11—C16	121.1 (2)	O8—S1—O6	108.43 (9)
C12—C11—S2	119.64 (17)	O7—S1—C18	111.86 (11)
C16—C11—S2	119.27 (17)	O8—S1—C18	109.96 (10)
C13—C12—C11	118.8 (2)	O6—S1—C18	98.69 (8)
C13—C12—H12	120.6	O5—S2—O4	119.21 (12)
C11—C12—H12	120.6	O5—S2—O3	107.16 (10)
C12—C13—C14	121.7 (2)	O4—S2—O3	107.48 (10)
C12—C13—H13	119.2	O5—S2—C11	110.58 (10)
C14—C13—H13	119.2	O4—S2—C11	111.42 (11)
C13—C14—C15	117.8 (2)	O3—S2—C11	98.85 (9)

### Hydrogen-bond geometry (Å, º)

**Table:**

*D*—H···*A*	*D*—H	H···*A*	*D*···*A*	*D*—H···*A*
C7—H7···O4^i^	0.93	2.48	3.333 (3)	153
C3—H3···O8^ii^	0.93	2.49	3.245 (3)	139

Symmetry codes: (i) *x*−1, *y*, *z*; (ii) −*x*+1, −*y*, −*z*+1.
